# Synthesis of ethyl acetate from glucose by *Kluyveromyces marxianus*, *Cyberlindnera jadinii* and *Wickerhamomyces anomalus* depending on the induction mode

**DOI:** 10.1002/elsc.202000048

**Published:** 2020-12-23

**Authors:** Andreas Hoffmann, Christian Kupsch, Thomas Walther, Christian Löser

**Affiliations:** ^1^ Chair of Bioprocess Engineering Institute of Natural Materials Technology Technische Universität Dresden Dresden Germany

**Keywords:** acetaldehyde, bioreactor cultivation, ethanol, iron limitation, oxygen limitation

## Abstract

Ethyl acetate is currently produced from fossil carbon resources. This ester could also be microbially synthesized from sugar‐rich wastes of the food industry. Wild‐type strains with GRAS status are preferred for such applications. Production of ethyl acetate by wild‐type yeasts has been repeatedly reported, but comparative studies with several strains at various induction modes are largely missing. Here, synthesis of ethyl acetate by three yeasts with GRAS status, *Kluyveromyces marxianus* DSM 5422, *Cyberlindnera jadinii* DSM 2361 and *Wickerhamomyces anomalus* DSM 6766, was studied under identical and well‐defined conditions in an aerated bioreactor, by inducing the ester synthesis via iron or oxygen limitation. Balancing the ester synthesis was based on measured concentrations of ethyl acetate in the exhaust gas, delivering masses of synthesized ester and synthesis rates in a high temporal resolution. All tested yeasts synthesized ethyl acetate under these conditions, but the intensity varied with the strain and induction mode. The highest yields were achieved under iron limitation with *K. marxianus* (0.182 g g^–1^) and under oxygen limitation with *W. anomalus* (0.053 g g^–1^). Iron limitation proved to be the better inducer for ester synthesis while oxygen limitation favored ethanol formation. *K. marxianus* DSM 5422 was the most potent producer of ethyl acetate exhibiting the highest biomass‐specific synthesis rate of 0.5 g g^–1^h^–1^ under moderate iron limitation.

**Abbreviations**
AATs: alcohol acyltransferases
Eat1: ethanol acetyltransferase
ETC: electron transport chain

## 1 INTRODUCTION

Ethyl acetate is, due to its microbial degradability [1, 2], rated as an environmentally friendly organic solvent with many industrial applications [3]. This ester is currently produced from fossil resources such as natural gas and crude‐oil constituents by energy‐intensive petrochemical processes [4, 5], although microbial synthesis of ethyl acetate from renewable resources exhibits a high economical potential [6]. Ethyl acetate along with other esters is formed by yeasts as an aroma compound during fermentative production of beverages and other foodstuffs [7, 8]. Some yeasts produce ethyl acetate in high amounts whereby *Cyberlindnera jadinii*, *Kluyveromyces marxianus* and *Wickerhamomyces anomalus* have been turned out as the most potent species (see [5] and Supporting Information).

Microbial synthesis of ethyl acetate in yeasts has been associated with three enzyme‐catalyzed reactions [5, 9, 10, 11]: (1) esterification of ethanol and acetate by reverse esterase activity, (2) dehydrogenation of 1‐ethoxyethanol, a spontaneously formed adduct from acetaldehyde and ethanol, by hemiacetal dehydrogenase activity as a side activity of some alcohol dehydrogenases, and (3) condensation of the acetyl moiety of acetyl‐CoA with ethanol by alcohol acyltransferases (AATs). Formation of ethyl acetate by esterases in yeasts is unlikely since the aqueous conditions in cells strongly favor ester hydrolysis over ester synthesis [11]. Some formation of ethyl acetate via hemiacetal dehydrogenation has been described for *Saccharomyces cerevisiae* [12], *C. jadinii* [13] and *K. marxianus* [14]. But synthesis of ethyl acetate by AATs has been proven as the most important pathway in yeasts. Kruis et al. [10, 11] have given comprehensive reviews of AATs whereupon acetate esters are synthesized in yeasts by acetyltransferases (Aft1 and Aft2) and the recently identified ethanol acetyltransferase 1 (Eat1). Eat1 catalyzes bulk synthesis of ethyl acetate in many yeasts such as *W. anomalus*, *W. ciferrii*, *K. marxianus*, *K. lactis*, *C. jadinii* and *C. fabianii* while Aft1 and Aft2 seem to play only a minor role for synthesis of ethyl acetate in these yeasts [10, 15]. The Eat1 enzyme also exhibits some esterase activity (hydrolysis of ethyl acetate) and thioesterase activity (hydrolysis of acetyl‐CoA) [10].

Intensive synthesis of ethyl acetate is caused by suboptimal growth conditions such as iron limitation [14, 16–28], copper limitation [24], and oxygen limitation [10, 17, 19, 20, 29, 30]. In detail, a lack of intracellular iron diminishes the activity of aconitase, succinate dehydrogenase and the electron transport chain (ETC) since all of them depend on iron [23]. Lacking copper reduces the activity of ETC complex IV [24]. A limited availability of oxygen as the terminal electron acceptor reduces the ETC activity as well [30]. The slowed‐down re‐oxidation of NADH in the ETC results in lacking mitochondrial NAD^+^ which in turn, together with a reduced aconitase and succinate dehydrogenase activity under iron limitation, diminishes the flux of acetyl‐CoA into the Krebs cycle [15, 23, 30]. Acetyl‐CoA is lastly diverted to synthesis of ethyl acetate to counteract accumulation of intra‐mitochondrial acetyl‐CoA [15, 16, 28, 30]. This concept is supported by the observation that ETC inhibitors initiate synthesis of ethyl acetate in *K. marxianus* [30] and by the recent finding that Eat1 is localized in the mitochondria [15, 28].

**PRACTICAL APPLICATION**

Recent identification of the Eat1 enzyme responsible for bulk synthesis of ethyl acetate in yeasts from sugar inspires development of efficient production strains by means of genetic engineering. In the food industry, however, wild‐type strains with GRAS status are preferred to convert sugar‐rich wastes into valuable products. Therefore, three wild‐type yeasts were compared regarding the potential for efficient synthesis of ethyl acetate. The ester synthesis was induced by a limited availability of iron or oxygen. Uniform cultivation conditions allowed reliable comparison of the selected strains.

Identification of Eat1 as the key enzyme for bulk synthesis of ethyl acetate and the knowledge that Eat1 is localized in the mitochondria boost the development of genetically modified microbes for optimized ethyl acetate production from sugars [11, 15, 31, 32]. However, microbial conversion of sugar‐rich wastes from the food industry into valuable products (i.e. production of ethanol and ethyl acetate from whey) is preferably conducted by wild‐type yeast strains with GRAS status for a positive company perception and because of a lacking customer acceptance for genetically modified microorganisms. This means that wild‐type strains will continue to play an important role as potential producers of ethyl acetate in this field.

Evaluation of microbial ethyl acetate production has usually been based on the average ester yield of the total process (mass of ester synthesized divided by the mass of sugar consumed, $Y_{EA/S}$). However, there are additional assessment criteria for economic process evaluation such as the productivity ($R_{EA}$), the biomass‐specific synthesis rate ($r_{EA}$), and the product selectivity, which have not yet received much attention. This situation possibly results from the high effort for determination of these parameters due to the volatility of ethyl acetate [22, 26, 27]. Calculation of the $R_{EA}$ and $r_{EA}$ variables was hitherto based on analyses of both the dissolved and stripped ester [22, 23, 25, 26, 28, 32]. A new less labor‐intensive approach for balancing volatile organic compounds (VOCs) has therefore been developed which is solely based on repeatedly measured ester concentrations in the exhaust gas of aerated bioreactors [33]. This new method utilizes the correlation between the liquid and gas‐phase concentrations of VOCs with the partition coefficient as the proportionality factor. This principle has already been applied to ethyl acetate, ethanol and acetaldehyde [33].

Comparative studies of bulk synthesis of ethyl acetate by various yeast species and/or different induction modes, performed under uniform and well‐defined experimental conditions, are rare. Formation of ethyl acetate as an aroma compound by various wine yeasts has been repeatedly compared, but the amounts of synthesized ethyl acetate were generally low [29, 34, 35]. Löser et al. [21] have examined the bulk production of ethyl acetate from whey by diverse *K. marxianus* and *K. lactis* strains, but induction of ester synthesis via a lack of iron was superimposed by unwanted oxygen limitation. Wu et al. [36] have studied the synthesis of ethyl acetate by diverse yeast species from various sugars, but the induction mode remained unexplained and the product titers were presented only semi‐quantitatively in form of heatmaps. Ravasio et al. [8] have analyzed the synthesis of ethyl acetate by many yeast strains, but the cultivation conditions were not clearly defined and only qualitative results were given. Lastly, Kruis et al. [28] have compared the synthesis of ethyl acetate by diverse yeast species in well‐defined iron‐limited chemostat cultures combined with reliable detection of ester synthesis, but product formation was only specified by yields.

Here, we compare microbial synthesis of ethyl acetate by three wild‐type yeast species with GRAS status, *K. marxianus*, *C. jadinii* and *W. anomalus*. The cultivations were conducted in well‐defined and largely identical aerobic batch processes using a 1‐L stirred bioreactor and glucose‐based mineral medium. Synthesis of ethyl acetate was induced by limitation of iron or oxygen. Additional cultivations without induction served as a reference. Periodic measurement of the stripped volatiles enables calculation of reaction rates in a high temporal resolution, allowing a deeper insight into the process of synthesis of ethyl acetate and other VOCs. Such comparison of ester synthesis under uniform conditions with the induction mode as the varied parameter is presented here for the first time.

## 2 MATERIALS AND METHODS

### 2.1 Microorganism and culture medium

*Kluyveromyces marxianus* DSM 5422, *Cyberlindnera jadinii* DSM 2361 and *Wickerhamomyces anomalus* DSM 6766 originate from the Deutsche Sammlung von Mikroorganismen und Zellkulturen GmbH (Braunschweig, Germany). Inocula were obtained by one‐day cultivation on yeast extract‐glucose‐chloramphenicol agar (YGC agar, Roth GmbH, Germany) at 32°C.

For bioreactor cultivations, a glucose‐based mineral medium was used that fulfilled the nutritional requirements of the three yeast species. For preparing 1 L medium, an autoclaved 1‐L Schott bottle was filled with sterile macro‐mineral solution, glucose stock solution, vitamin solution and trace‐element solution and was filled up to 1 L with sterile water as described in [33]. The ready‐to‐use medium contained the following constituents per liter: 20 g glucose, 5 g (NH_4_)_2_SO_4_, 3 g KH_2_PO_4_, 0.5 g MgSO_4_·7 H_2_O, 0.1 g NaCl, 25 mg inositol, 5 mg FeSO_4_·7 H_2_O, 5 mg ZnSO_4_·7 H_2_O, 1 mg Ca pantothenate, 1 mg nicotinic acid, 1 mg thiamine·HCl, 1 mg pyridoxine·HCl, 1 mg CaCl_2_·2 H_2_O, 1 mg CuSO_4_·5 H_2_O, 1 mg MnSO_4_·4 H_2_O, 0.2 mg CoSO_4_·7 H_2_O, 0.2 mg H_3_BO_3_, 0.2 mg 4‐aminobenzoate, 0.2 mg riboflavin, 0.2 mg folic acid, 0.1 mg Na_2_MoO_4_·2 H_2_O, 0.1 mg NiSO_4_·6 H_2_O, 0.1 mg KI, and 0.05 mg biotin. In case of iron‐limitation experiments, iron sulfate was omitted from the medium.

### 2.2 Bioreactor cultivation

All cultivations were conducted in a 1‐L stirred bioreactor, mixed by three six‐bladed Rushton turbines and controlled by an ADI 1030 Biocontroller (Applikon) as described previously [22]. The here applied cultivation procedure was described in [33]. The reactor was charged with 0.3 mL Antifoam A (Fluka), autoclaved for 20 min at 121°C, internally dried by a sterile air flow, and filled with 600 mL culture medium. The pH value of the medium was heightened to 6 by addition of a small volume 2 M NaOH. The medium was inoculated with one inoculating loop of biomass from an agar‐plate culture, resulting in an initial cell concentration of 0.0006 g L^–1^. The reactor was operated at a temperature of 32°C, a stirrer speed of 1200 rpm, a pH of ≥5 (the controlled supply of 2 M NaOH avoided the drop of pH below 5 as a result of ammonium consumption, while supply of acid was not required since the pH never exceeded a value of 6), and an air flow of 30 L h^–1^ (given for 273.15 K and 101,325 Pa). Oxygen limitation was brought about by an abrupt stirrer‐speed reduction (details below). Oxygen consumption and CO_2_ formation was determined by measuring the O_2_ and CO_2_ content in the exhaust gas by an EL3020 gas analyzer (ABB, Germany). Regular sampling started around 10 h after inoculation. Liquid samples taken at 1‐h intervals were used for $OD_{600nm}$ measurements and processed for analyses of biomass, glucose and dissolved metabolites according to Urit et al. [22]. VOCs in the exhaust gas were analyzed at 15‐min intervals as described in [22]. Specific growth rates were derived from measured $OD_{600nm}$ values, cumulative O_2_ consumption and cumulative CO_2_ formation [33].

### 2.3 Analyses

The $OD_{600nm}$ of cell suspensions was measured with a DU 520 photometer (Beckman). The biomass dry weight was determined by separating yeast cells from the suspension via centrifugation, washing the pellet twice with deionized water and drying at 105°C until weight constancy. Glucose was determined by the DNS method according to the procedure of Hortsch et al. [37]. VOCs in liquid and gas samples were analyzed by gas chromatography [22]. Acetate, glycerol and other non‐volatile metabolites were quantified by high‐performance liquid chromatography [38]. Confidence intervals of data were calculated for a confidence level of 95%.

### 2.4 Balancing the synthesis of volatile metabolites

Balancing the ester synthesis in bioprocesses has so far been based on ester concentrations measured in both the liquid and gas phase [5, 22–26, 28, 32]. Here, another approach was applied which only requires repeatedly measured gas‐phase concentrations of the VOCs of interest. Essential advantages of this alternative method are a higher precision, a better temporal resolution and time savings. The following equations were used for calculating reaction rates and masses of formed VOCs [33]:

(1) $$\begin{array}{ccc} R_{\mathrm{VOC}}\left(t\cdots t+\Delta t\right) & = & \frac{C_{\mathrm{VOC},G}\left(t+\Delta t\right)-C_{\mathrm{VOC},G}\left(t\right)}{\Delta t}\cdot \frac{K_{\mathrm{VOC},L/G}}{1-\beta_{\mathrm{VOC}}}\\ & & \cdot \frac{F_{G}}{F_{G,R}}+C_{\mathrm{VOC},G}\left(t\cdots t+\Delta t\right)\cdot \frac{F_{G}}{V_{L}} \end{array}$$

(2) $$r_{VOC}(t\ldots t+\Delta t)\;=\frac{R_{VOC}\left(t\ldots t+\Delta t\right)}{C_{X}\left(t\ldots t+\Delta t\right)}\;$$

(3) $$\begin{array}{c} m_{VOC}\left(t\right)=\int_{\tau =0}^{\tau =t}C_{VOC,G}\left(\tau \right)\cdot F_{G}\cdot d\tau \;+C_{VOC,G}\left(t\right)\\ \;\;\;\;\;\cdot \frac{K_{VOC,L/G}}{1-\beta_{VOC}}\cdot \frac{F_{G}}{F_{G,R}}\cdot V_{L} \end{array}$$

These calculations require the following parameters: the repeatedly measured VOC content in the exhaust gas ($C_{VOC,G}$, analyzed at 15‐min intervals), the biomass concentrations ($C_{X}$, measured at 1‐h intervals and interpolated by a cubic smoothing procedure), the liquid volume ($V_{L}$, temporal changes taken into account according to [39]), the gas flows at the gas‐line exit and at the bioreactor outlet ($F_{G}$ and $F_{G,R}$, their temporal variations calculated according to [33]), the partition coefficients ($K_{VOC,L/G}$), and the retention efficiency of the condenser for VOCs ($\beta_{VOC}$). The $K_{VOC,L/G}$ and $\beta_{VOC}$ parameters have been determined in stripping experiments for this specific cultivation system, the used medium and the applied process parameters [33]: $K_{EA,L/G}$ = 92.3 L L^–1^ and $\beta_{EA}$ ≈ 0 for ethyl acetate, $K_{AA,L/G}$ = 194.5 L L^–1^ and $\beta_{AA}$ = 0.0146 for acetaldehyde, and $K_{EtOH,L/G}$ = 2430 L L^–1^ and $\beta_{EtOH}$ = 0.147 for ethanol.

Partition coefficients are influenced by the concentration of dissolved compounds such as sugar and inorganic salts [21, 22, 33] and can thus change during the cultivation process. Sugar consumption increases and NaOH dosage for pH correction decreases the partition coefficients. However, previous cultivation experiments have clearly shown that the actual $K_{VOC,L/G}$ variation over time is insignificant (for details it is referred to [33]).

The volume‐specific reaction rate $R_{VOC}$ is a process‐related parameter and represents the mass of formed or consumed VOC per hour and liter culture volume. The biomass‐specific reaction rate $r_{VOC}$ is a biological parameter indicating the mass of synthesized or metabolized VOC per gram biomass and hour. In this work, synthesis or consumption rates represent excretion or uptake rates of the considered compounds but not intracellular reaction rates. Positive $R_{VOC}$ and $r_{VOC}$ values correspond to VOC synthesis, while negative values mean VOC utilization. The cumulatively formed mass of a VOC at a given time $m_{VOC}\left(t\right)$ is the sum of the already stripped and currently dissolved VOC [22]. During VOC synthesis, $m_{VOC}\left(t\right)$ temporally increases, whereas at VOC degradation, $m_{VOC}\left(t\right)$ decreases. Average yields for the total process were calculated from maximum $m_{VOC}\left(t\right)$ values divided by the mass of glucose consumed.

## 3 RESULTS AND DISCUSSION

Here, it is reported on synthesis of ethyl acetate by selected strains of *K. marxianus*, *C. jadinii*, and *W. anomalus* during cultivation under uniform conditions and variable induction modes. These three yeast species are known for their potential of ethyl acetate synthesis (Supporting Information). Synthesis of ethyl acetate was induced by a deficit of iron or oxygen, and was also studied under non‐induced conditions as a reference. Uniform cultivation conditions combined with data analysis in a high temporal resolution allowed comparison regarding synthesis rates, yields, and a possible re‐uptake of excreted volatile metabolites like ethyl acetate, ethanol and acetaldehyde.

All processes were performed in a 1‐L stirred bioreactor using 0.6 L mineral medium with 20 g L^–1^ glucose at 32°C, pH ≥ 5, and aeration with 30 L h^–1^. Further varied cultivation conditions are explained below.

### 3.1 Reference processes

All reference experiments were performed with surplus iron. Intensive stirring at 1200 rpm and vigorous aeration ensured aerobic conditions during the entire cultivation period (DO always ≥40% air saturation). Yeast growth was respiratory and only restricted by available glucose. The respiration quotient (RQ) of these processes was therefore around one as expected (Table 1).

**TABLE 1:** Parameters of cell growth, sugar consumption, and product synthesis during the aerobic batch cultivation of *K. marxianus* DSM 5422 (cultivations *A–C*), *C. jadinii* DSM 2361 (*D–F*) and *W. anomalus* DSM 6766 (*G to I*) in a 1‐L stirred bioreactor using 0.6 L glucose‐based mineral medium under varied cultivation conditions; Reference processes without limitation (*A, D*, and *G*), processes with iron limitation (*B, E*, and *H*), and processes with oxygen limitation (*C, F*, and *I*); Cultivation conditions as stated in the legends of Figures 1, 2, 3; n. i. means not identifiable, n. d. means not determined

	Cultivation process								
Parameter	*A*	*B*	*C*	*D*	*E*	*F*	*G*	*H*	*I*
Strain	*K. marxianus* DSM 5422			*C. jadinii* DSM 2361			*W. anomalus* DSM 6766		
Iron supplementation?	Yes	No	Yes	Yes	No	Yes	Yes	No	Yes
Iron limitation?	No	Yes	No	No	Yes	No	No	Yes	No
Oxygen limitation?	No	No	Yes	No	No	Yes	No	No	Yes
$\mu_{max}$ calculated from CO_2_ formation [h^–1^]	0.614	0.615	0.617	0.619	0.618	0.617	0.418	n. d.	0.410
$\mu_{max}$ calculated from O_2_ consumption [h^–1^]	0.615	0.621	0.610	0.617	0.601	0.614	0.410	n. d.	0.411
$\mu_{max}$ calculated from *OD_600nm_* [h^–1^]	0.600	0.612	0.619	0.617	0.598	0.618	0.416	0.415	0.410
Consumed glucose [g]	11.65	11.68	11.62	11.63	11.59	11.56	11.35	11.31	11.65
Final biomass concentration [g L^–1^]	9.52	4.53	7.02	9.90	7.70	9.61	10.81	7.08	9.55
Average RQ [mol mol^–1^]	1.03	1.17	1.07	1.02	1.05	1.06	1.03	1.07	1.10
Maximum $C_{EA,G}$ [mg L^–1^]	2.09	12.83	4.97	0.00	1.60	2.95	0.01	2.50	6.31
Maximum $C_{EA,L}$ [g L^–1^]	0.19	1.18	0.46	0.00	0.15	0.27	0.00	0.23	0.58
Mass of formed ethyl acetate, $m_{EA}$ [g]	0.28	2.13	0.49	0.00	0.33	0.25	0.00	0.52	0.61
Maximum $R_{EA}$ [g L^–1^h^–1^]	0.18	0.86	0.56	0.00	0.11	0.45	0.00	0.16	0.63
Maximum $r_{EA}$ [mg g^–1^h^–1^]	38	502	145	0	34	53	0	44	112
Minimum $r_{EA}$ [mg g^–1^h^–1^]	‒1	‒2	‒3	0	‒1	‒17	0	‒8	‒13
Overall yield of ethyl acetate, $Y_{EA/S}$ [g g^–1^]	0.024	0.182	0.042	0.000	0.028	0.021	0.000	0.046	0.053
$Y_{EA/S}$‐$Y_{EA/S,max}$ ratio [%]	4.9	37.2	8.6	0.0	5.7	4.3	0.0	9.4	10.8
Selectivity of ester formation [g g^–1^]	0.87	0.53	0.20	n. i.	0.23	0.11	n. i.	0.38	0.27
Maximum $C_{EtOH,G}$ [mg L^–1^]	0.03	0.92	0.96	0.00	0.02	1.15	0.02	0.01	0.93
Maximum $C_{EtOH,L}$ [g L^–1^]	0.07	2.47	2.60	0.00	0.06	3.10	0.05	0.04	2.51
Mass of formed ethanol, $m_{EtOH}$ [g]	0.04	1.52	1.52	0.00	0.04	1.82	0.03	0.02	1.48
Maximum $R_{EtOH}$ [g L^–1^h^–1^]	0.07	0.93	2.08	0.00	0.06	2.60	0.06	0.03	1.52
Maximum $r_{EtOH}$ [mg g^–1^h^–1^]	23	262	358	0	33	424	29	37	244
Minimum $r_{EtOH}$ [mg g^–1^h^–1^]	‒8	‒47	‒51	0	‒10	‒168	‒7	‒5	‒88
Overall yield of ethanol, $Y_{EtOH/S}$ [g g^–1^]	0.004	0.130	0.131	0.000	0.003	0.158	0.003	0.002	0.127
Maximum $C_{AA,G}$ [mg L^–1^]	0.00	0.93	1.30	0.00	0.00	0.14	0.00	0.01	0.18
Mass of formed acetaldehyde, $m_{AA}$ [g]	0.00	0.19	0.27	0.00	0.00	0.02	0.00	0.00	0.03
Maximum $R_{AA}$ [g L^–1^h^–1^]	0.00	0.21	0.22	0.00	0.00	0.03	0.00	0.00	0.02
Maximum $r_{AA}$ [mg g^–1^h^–1^]	0	47	34	0	0	6	0	1	6
Minimum $r_{AA}$ [mg g^–1^h^–1^]	0	n. i.	n. i.	0	0	‒2	0	0	‒7
Overall yield of acetaldehyde, $Y_{AA/S}$ [g g^–1^]	0.000	0.016	0.024	0.000	0.000	0.002	0.000	0.000	0.002

The growth of the three studied yeasts was exponential with a constant growth rate until glucose became a limiting factor (shortly before sugar depletion). Maximum specific growth rates were determined for the exponential growth phase based on $OD_{600nm}$ and exhaust‐gas data, yielding highly consistent $\mu_{max}$ values (Table 1). *K. marxianus* and *C. jadinii* grew with nearly the same rate ($\mu_{max}$ = 0.610 and 0.618 h^–1^) while *W. anomalus* grew distinctly slower ($\mu_{max}$ = 0.415 h^–1^). The slower growth of *W. anomalus* reasoned a longer‐lasting process.

**FIGURE 1:**
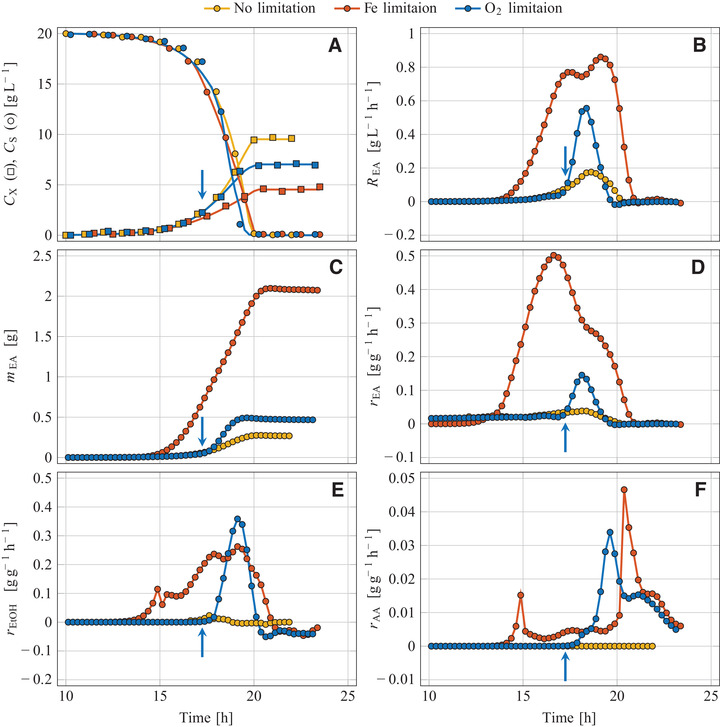
Aerobic batch cultivation of *K. marxianus* DSM 5422 in glucose‐based mineral medium at various process regimes; Non‐limited reference process *A* (medium with FeSO_4_, stirrer speed 1200 rpm), iron‐limited process *B* (medium without FeSO_4_, 1200 rpm), oxygen‐limited process *C* (medium with FeSO_4_, initiation of O_2_ limitation by reducing the stirrer speed from 1200 to 600 rpm as indicated by arrows); (A) Sugar and biomass concentration, (B) volume‐specific reaction rate of ethyl acetate, (C) mass of formed ethyl acetate, and biomass‐specific reaction rates of (D) ethyl acetate, (E) ethanol and (F) acetaldehyde

**FIGURE 2:**
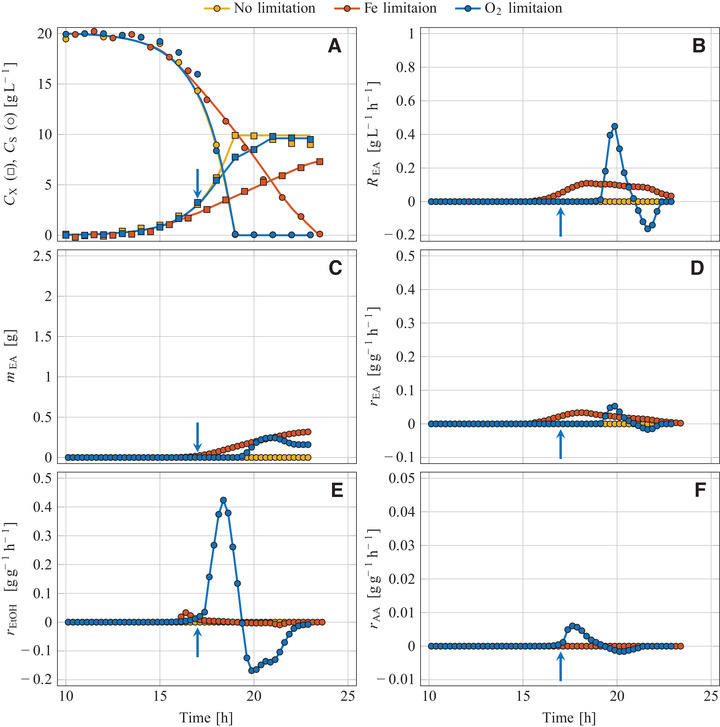
Aerobic batch cultivation of *C. jadinii* DSM 2361 in glucose‐based mineral medium at various process regimes; Non‐limited reference process *D* (medium with FeSO_4_, stirrer speed 1200 rpm), iron‐limited process *E* (medium without FeSO_4_, 1200 rpm), oxygen‐limited process *F* (medium with FeSO_4_, initiation of O_2_ limitation by reducing the stirrer speed from 1200 to 600 rpm as indicated by arrows); (A) Sugar and biomass concentration, (B) volume‐specific reaction rate of ethyl acetate, (C) mass of formed ethyl acetate, and biomass‐specific reaction rates of (D) ethyl acetate, (E) ethanol and (F) acetaldehyde

**FIGURE 3:**
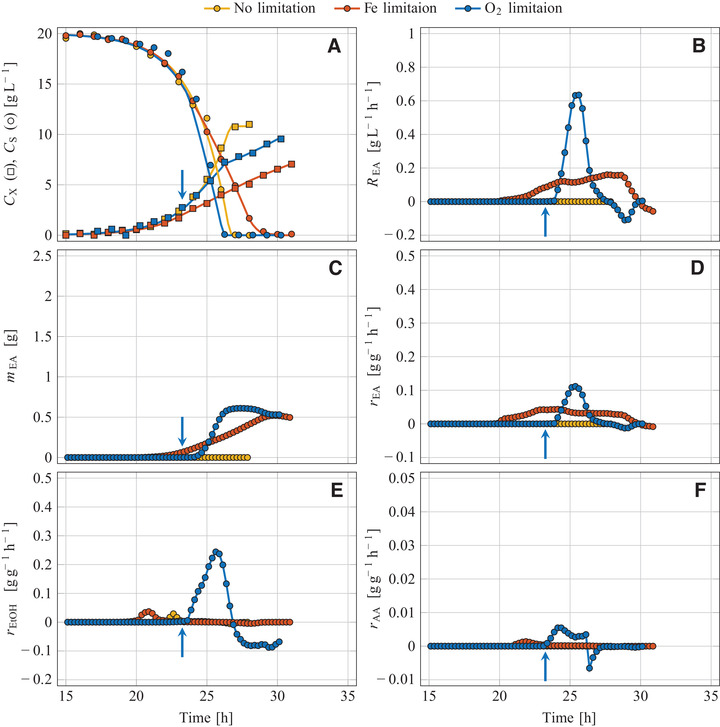
Aerobic batch cultivation of *W. anomalus* DSM 6766 in glucose‐based mineral medium at various process regimes; Non‐limited reference process *G* (medium with FeSO_4_, stirrer speed 1200 rpm), iron‐limited process *H* (medium without FeSO_4_, 1200 rpm), oxygen‐limited process *I* (medium with FeSO_4_, initiation of O_2_ limitation by reducing the stirrer speed from 1200 to 500 rpm as indicated by arrows); (A) Sugar and biomass concentration, (B) volume‐specific reaction rate of ethyl acetate, (C) mass of formed ethyl acetate, and biomass‐specific reaction rates of (D) ethyl acetate, (E) ethanol and (F) acetaldehyde

Glucose was used for growth (Figures 1A, 2A, and 3A) but not for producing VOCs (Figures 1, 2, 3 and $Y_{VOC/S}$ values in Table 1). Only traces of ethanol were formed which is explained by the Crabtree‐negative nature of *K. marxianus* [40, 41, 42, 43], *C. jadinii* [40, 41, 44], and *W. anomalus* [45, 46]. Some ethyl acetate was formed by *K. marxianus* DSM 5422 with a maximum rate of 38 mg g^–1^h^–1^, resulting in an overall yield of $Y_{EA/S}$ = 0.024 g g^–1^. This ester production is interpreted as a basal synthesis and confirms previous observations [22, 23, 24].

### 3.2 Iron‐limited processes

Iron limitation as a trigger for bulk synthesis of ethyl acetate has been known for a long time. The ester is formed from sugar by *C. jadinii*, *C. fabianii*, *K. marxianus*, *K. lactis* and *W. anomalus* under iron limitation while surplus iron represses ester formation [18, 19, 22–24, 28, 47].

In iron‐limited experiments, FeSO_4_ was omitted from the medium, but a small amount of iron was provided by impurities of media constituents (studied in detail by Thomas et Dawson [16]). Shake‐flask experiments demonstrated that these traces of iron allow some yeast growth (not shown). Dissolution of some more iron from the stainless steel fittings of the bioreactor cannot be definitely excluded (such iron dissolution has been observed during bioreactor cultivation of *K. marxianus* at an elevated temperature of 42°C [27]).

The strongly limited availability of iron resulted in a reduced growth of all strains compared to the reference experiments (Figures 1A, 2A, and 3A). The initial growth phase was exponential, based on the mentioned iron impurities, and exhibited the same growth rates as found in the reference experiments (Table 1). After depletion of iron in the culture medium, the intracellular iron content decreased steadily due to biomass expansion at continued growth (for details see [39]). The diminishing iron content of the yeast cells resulted in a decreasing growth rate with a shift from exponential to linear growth (Figures 1A, 2A, and 3A). Such a μ reduction under iron‐limited conditions has already been observed during cultivation of *K. marxianus* DSM 5422 in whey‐based media [21, 22]. Here, the period of iron‐limited sugar utilization lasted 7 to 9 h.

The decreasing iron content of the yeasts also triggered synthesis of metabolites, predominantly ethyl acetate. The measured exhaust‐gas concentrations of ethyl acetate, ethanol and acetaldehyde were used to calculate $R_{VOC}\left(t\right)$, $r_{VOC}\left(t\right)$ and $m_{VOC}\left(t\right)$ values for these metabolites via Equations (1–3).

$R_{VOC}\left(t\right)$ and $m_{VOC}\left(t\right)$ were exemplarily depicted for ethyl acetate (Figures 1, 2, 3). The $R_{EA}\left(t\right)$ variables increased after induction of ester synthesis, later decreased due to a diminishing synthesis rate, and became zero after glucose depletion. The courses of $m_{EA}\left(t\right)$ progressively increased over time; a later decrease of $m_{EA}\left(t\right)$ due to microbial degradation of dissolved ester was detected for *W. anomalus* but was not observed for *K. marxianus* and *C. jadinii*.

The intensity of metabolite synthesis was evaluated with the biomass‐specific synthesis rates ($r_{VOC}\left(t\right)$ in Figures 1, 2, 3, maximum and minimum $r_{VOC}$ values in Table 1). All tested yeasts formed ethyl acetate under iron‐limited conditions (Table 1), but *K. marxianus* DSM 5422 exhibited by far the most intensive ester synthesis. Its maximum $r_{EA}$ value of 502 mg g^–1^h^–1^ is in line with earlier observations [23, 25]. According to the literature, *K. marxianus* seems to be the most potent producer of ethyl acetate at least with regard to the synthesis rate (for published data see Supporting Information).

The overall yields of ethyl acetate ($Y_{EA/S}$) in these experiments were comparatively low (Table 1) due to the non‐optimized relation between the available iron and sugar. Too much iron compared to the usable sugar promotes yeast growth and reduces ester synthesis (for details see [23, 25]). *K. marxianus* exhibited the highest yield ($Y_{EA/S}$ = 0.182 g g^–1^, corresponding to 37.2% of the theoretical maximum) compared to *C. jadinii* and *W. anomalus* ($Y_{EA/S}$ = 0.028 and 0.046 g g^–1^, respectively).

In earlier studies, *K. marxianus* and *C. jadinii* strains have exhibited similar yields, but *W. anomalus* strains often have been less effective (Supporting Information). The cultivation conditions in various studies have been quite different so that reliable comparison is not possible. Only one comparative study is known to us, where several yeasts have been cultivated in a iron‐limited chemostat under uniform process conditions [28]: the ester yield of *W. anomalus* DSM 6766 was highest (0.17 g g^–1^), the yield of *K. marxianus* DSM 5422 was somewhat lower (0.14 g g^–1^), and *C. jadinii* CECT 1946 produced least ester (0.10 g g^–1^). The discrepancy to our observations suggests that specific cultivation conditions such as culture medium, cultivation mode, growth rate and degree of iron limitation take influence on the observed yields.

Iron limitation also initiated ethanol synthesis in *K. marxianus* (Figure 3C). Aerobic formation of ethanol from sugar by Crabtree‐negative yeasts is caused by growth‐inhibiting substances or a specific nutrient limitation [48], here induced by iron limitation. However, ethanol formation started later than ester synthesis (compare $r_{EA}\left(t\right)$ and $r_{EtOH}\left(t\right)$ in Figures 1D and 1E). Moderate iron limitation favored ester formation while severe iron limitation at a later period intensified ethanol synthesis. The two other yeasts *C. jadinii* and *W. anomalus* formed only traces of ethanol under iron‐limited conditions (Figures 2E and 3A, Table 1). Simultaneous synthesis of ethanol and ethyl acetate has been frequently observed [19, 22–28]. Ethanol as a by‐product is undesirable since ethanol synthesis wastes sugar and pollutes the produced ester.

Acetaldehyde as another volatile by‐product was formed under iron‐limited conditions by *K. marxianus* but not by the two other yeasts. However, the synthesis rate and the formed amount were quite low, and the main quantity was formed from ethanol after sugar depletion (Figure 1F, Table 1). Synthesis of acetaldehyde was also observed during cultivation of *K. marxianus* in iron‐deficient whey‐based medium [19]. Acetaldehyde has become even the main product during cultivation of *C. jadinii* with ethanol as the substrate [49].

Acetate was synthesized by *C. jadinii* and *W. anomalus* under iron‐limited conditions from glucose (maximum values of 1.7 and 1.4 g L^–1^ at the moment of sugar depletion) while *K. marxianus* produced acetate only in traces. Acetate is not volatile at pH ≥ 5 and hence was only detected in the liquid phase. Synthesis of acetate as a by‐product has been repeatedly observed during iron‐limited cultivation of *K. marxianus* [18, 24–26, 28] and other yeasts like *K. lactis*, *C. jadinii*, *C. fabianii* and *W. anomalus* [28]. Such acetate synthesis is possibly caused by the esterase and thioesterase side activity of the Eat1 enzyme resulting in hydrolysis of ethyl acetate into acetate and ethanol or hydrolysis of acetyl‐CoA into acetate and CoA [10].

### 3.3 Oxygen‐limited processes

Induction of ethyl acetate synthesis by a deficit of oxygen often happened unintentionally and even unknowingly, and has been investigated purposefully only in a few cases (for details see [5] and Supporting Information). Intended induction via oxygen limitation has been realized in various ways: (1) by a varied headspace/culture volume ratio in shake flasks [17, 50], (2) by a changed oxygen content of the supplied gas [10, 19], (3) by a varied gas flow [19, 45, 51], or (4) by a varied agitation speed in stirred bioreactors [19, 20, 30, 45, 52]. However, the achieved results often have been of limited significance for several reasons: (1) the ester synthesis has been initiated by a combined limitation of oxygen and iron [17, 19, 20, 52], (2) ethanol has been used as a substrate or co‐substrate [19, 20, 52], and (3) quantification of ester synthesis has been afflicted with pitfalls (ester losses by stripping have remained unnoticed [51] or have been handled in a wrong way [19, 20, 45, 52]).

For this reason, we studied the ester synthesis at oxygen limitation under well‐defined process conditions. In these experiments, the medium was supplemented with surplus iron to avoid iron limitation. When the biomass concentration approached a value of approximately 3 g L^–1^, the stirrer speed was abruptly reduced from 1200 to 600 rpm (*K. marxianus*, *C. jadinii*) or 500 rpm (*W. anomalus*) for reducing the oxygen‐transfer rate. The stirrer‐speed reduction caused a quick drop of the DO below 10% air saturation. Continued growth reduced the DO below 1% within 15 min at a maximum. After sugar depletion, the oxygen demand was reduced and the DO quickly increased. The period of oxygen limitation was relatively short (2 to 3 h) compared to the duration of the iron‐limited ester synthesis (7 to 9 h). The drop of the $k_{L}a$ value at the moment of stirrer‐speed reduction did not influence the VOC stripping (for details see [22, 53]).

In the initial non‐limited cultivation period, yeast growth was exponential and as fast as in the non‐limited period of the other experiments ($\mu_{max}$ data in Table 1; individual $\mu_{max}$ values were used to calculate averages for each strain over the three cultivation modes: 0.614 ± 0.004 h^–1^ for *K. marxianus* DSM 5422, 0.613 ± 0.005 h^–1^ for *C. jadinii* DSM 2361, and 0.413 ± 0.003 h^–1^ for *W. anomalus* DSM 6766). The abrupt stirrer‐speed reduction restricted the O_2_ transfer and hence caused a linear growth pattern (Figures 1A, 2A and 3A) and a steadily decreasing specific growth rate. After glucose depletion, further growth was detected for *C. jadinii* and *W. anomalus* based on the consumption of previously formed metabolites.

The sugar‐uptake rate $R_{S}$ was not influenced by the limited availability of oxygen (Figures 1A, 2A, and 3A). Synthesis of ethanol, ethyl acetate and acetaldehyde started immediately after DO approached 0%, with exception of the ester synthesis by *C. jadinii* (Figures 1, 2, 3). Ethyl acetate was formed by all three yeasts (see $m_{EA}$ and $Y_{EA/S}$ in Table 1). *K. marxianus* and *W. anomalus* formed ethyl acetate from glucose (Figures 1C and 3C) while *C. jadinii* synthesized ethyl acetate after sugar depletion (Figure 2C). Maximum $r_{EA}$ values of *K. marxianus* and *W. anomalus* were quite high under oxygen limitation but distinctly lower than the maximum $r_{EA}$ of *K. marxianus* during iron‐limited cultivation (Table 1).

The intensity of ethyl acetate synthesis strongly depended on the degree of oxygen limitation. Severe oxygen limitation at a stirrer‐speed reduction from 1200 to 100 rpm suppressed ester synthesis and favored ethanol production by *K. marxianus* DSM 5422 ($C_{EtOH,L}$ up to 5 g/L; experiment not shown). In a previous batch culture of *K. marxianus* DSM 5422 in whey‐based medium, the stirrer‐speed reduction from 1200 to 600 rpm has resulted in low ester but high ethanol formation [30] which is explained by severe oxygen limitation due to a higher cell concentration in this experiment. Obviously, the degree of oxygen limitation had a considerable influence on the product spectrum.

Oxygen limitation caused intensive ethanol synthesis in all three yeast strains ($r_{EtOH}$ in Figs. 1E, 2E, 3E; $Y_{EtOH/S}$ in Table 1). Oxygen limitation favored ethanol over ester production; the $m_{EtOH}$‐$m_{EA}$ ratio was always >1 g g^–1^, ranging from 2.4 to 7.3 g g^–1^ (Table 1). Initiation of fermentative sugar utilization with ethanol formation by lacking oxygen is generally observed in Crabtree‐negative yeasts [10, 17, 19, 20, 30, 40, 47, 54]. A shift from ester to ethanol synthesis has also been observed by Kallel‐Mhiri et al. [19] under intensified oxygen limitation. Oxygen limitation as an inducer for synthesis of ethyl acetate is thus less suited at least in batch processes since the degree of oxygen limitation is difficult to control.

Acetaldehyde was a minor product under oxygen‐limited conditions (Figures 1F, 2F, and 3F). *K. marxianus* synthesized acetaldehyde at first from glucose but later from ethanol ($m_{AA}$ = 0.41 g, maximum $r_{AA}$ = 47 mg g^–1^h^–1^) while *C. jadinii* and *W. anomalus* formed less of this substance (Table 1). Acetaldehyde as a by‐product of ethyl acetate synthesis at oxygen‐limited cultivation has been observed for *K. marxianus* by Kallel‐Mhiri et al. [19] and for *W. anomalus* by Gray [50].

### 3.4 Conversion of synthesized metabolites

Synthesized VOCs are not removed immediately with the exhaust gas but transiently accumulate in the culture medium [33]. Dissolved metabolites can be re‐utilized by yeasts which is usually recognized by the decrease of their liquid‐phase concentrations. This principle only works for non‐volatile compounds (e.g. for acetate and glycerol) while temporally declining concentrations of dissolved VOCs could be caused either by exclusive stripping or by a combined utilization and stripping. The way of data evaluation which is practiced here allows to differentiate between microbial utilization and stripping: actual VOC utilization is noticed from negative biomass‐specific reaction rates (Table 1) while pure stripping without such VOC utilization results in $r_{VOC}$ ≈ 0.

**FIGURE 4:**
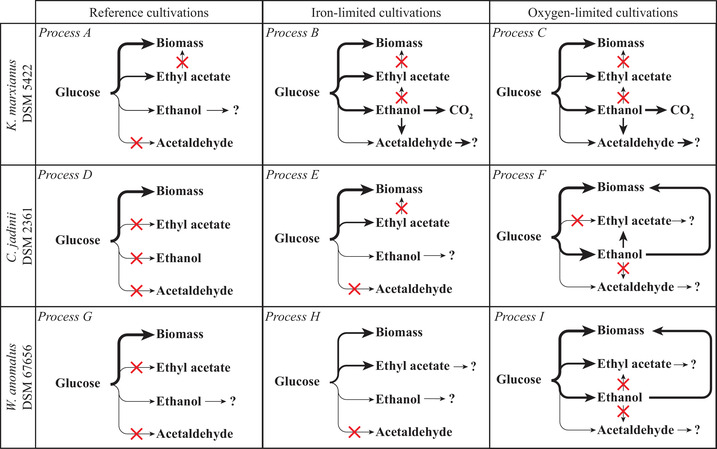
Visualization of the microbial conversion of glucose and formed metabolites by the three studied yeasts under the three cultivation conditions; line thicknesses of the arrows symbolize the masses of formed products in a logarithmic graduation; crossed out lines mean that the respective turnover did verifiably not occur; → ? means that the compound was microbially converted to an unknown product; process names correspond to the experiments in Figures 1, 2, 3 and Table 1

The microbial transformation of glucose into volatile metabolites and the further possible conversion of these metabolites is visualized in Figure 4; thick lines symbolize large metabolic fluxes from one compound to another, thin lines mean small fluxes, and crossed‐out lines stand for zero fluxes.

Ethyl acetate synthesized from glucose was later re‐absorbed by *W. anomalus* (minimum $r_{EA}$ = –13 mg g^–1^h^–1^), which has already been observed earlier for this yeast [54]. Such degradation of the ester is eventually facilitated by the esterase activity of the Eat1 enzyme [10, 32]. *K. marxianus* DSM 5422 synthesized much ethyl acetate from glucose but never utilized this ester again after sugar depletion ($r_{EA}$ did not became distinctly negative), and the observed decline of the ester concentration in the liquid and gas phase was solely caused by stripping. The absence of uptake of ethyl acetate by *K. marxianus* DSM 5422 has been repeatedly observed in the past even at high dissolved ester concentrations [25, 26]. This refers to a low ester‐hydrolyzing activity in this yeast which could be advantageous for its future application in large‐scale processes. Strain‐specific variations regarding the Eat1 side activities seem to exist which should be of interest for strain selection and strain development.

Ethanol synthesized from glucose was metabolized after sugar depletion (minimum $r_{EtOH}$ values in Table 1). *K. marxianus* DSM 5422 oxidized ethanol mainly to CO_2_ and some acetaldehyde (Fig. 4). Oxidation of ethanol to acetaldehyde by *C. jadinii* DSM 2361 did not occur although acetaldehyde has been observed even as the main product of *C. jadinii* at high ethanol concentrations [49]. Here, *C. jadinii* partially converted ethanol to ethyl acetate which has already been described in the literature [17, 52, 55]. Neither *K. marxianus* nor *W. anomalus* synthesized ethyl acetate from ethanol (Figure 4) although such a conversion has been repeatedly described for these yeasts [18–20, 51, 54].

### 3.5 Rates and yields of metabolite syntheses in relation to the specific growth rate

**FIGURE 5:**
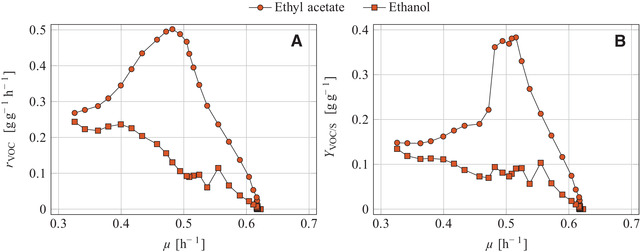
Aerobic batch cultivation of *K. marxianus* DSM 5422 in a 1‐L stirred bioreactor using 0.6 L glucose‐based mineral medium under iron‐limited conditions (medium without FeSO_4_); (A) Biomass‐specific reaction rates and (B) yields of ethyl acetate and ethanol depending on the specific growth rate; Time‐dependent reaction rates originate from Figure 1, and time‐dependent yields and specific growth rates were taken from [33]

In this study, the most intensive synthesis of ethyl acetate was observed under iron limitation for *K. marxianus* DSM 5422, exhibiting a maximum $r_{EA}$ value of 502 mg g^–1^h^–1^ (Table 1). The metabolite syntheses are therefore evaluated in more detail for this yeast under iron‐limited conditions. The biomass‐specific rate of ester synthesis was not constant but changed over time (Figure 1D) which has also been true for the yield of ethyl acetate and the specific growth rate as well [33]. The three parameters $r_{EA}\left(t\right)$, $Y_{EA/S}\left(t\right)$ and μ(t) were obviously governed by the degree of iron limitation or, more strictly speaking, by the intracellular iron content of the yeast cells ($x_{Fe}$). Several batch experiments have demonstrated that the growth rate and rate of ester synthesis by *C. jadinii* [47] and *K. marxianus* [23, 25] is influenced by the availability of iron. At a limited availability, iron uptake by the growing yeasts results in iron depletion in the medium, and continued growth based on stored iron let the intracellular iron content steadily decrease [39]. The diminishing iron content finally induces synthesis of ethyl acetate (for details on this mechanism it is referred to the Introduction chapter). In this study, the iron content of the yeast cells, $x_{Fe}\left(t\right)$, were not determined so that $r_{EA}$‐$x_{Fe}$ and $Y_{EA/S}$‐$x_{Fe}$ correlations can not be provided. The $r_{EA}\left(t\right)$ and $Y_{EA/S}\left(t\right)$ courses were therefore plotted against μ(t) together with the rates and yields of ethanol as an important by‐product (Figures 5A and 5B) since μ depends on $x_{Fe}$ as well.

The depicted $r_{EA}\left(\mu \right)$, $Y_{EA/S}\left(\mu \right),$ $r_{EtOH}\left(\mu \right)$ and $Y_{EtOH/S}\left(\mu \right)$ courses allow a deeper insight into the studied process. The $r_{EA}\left(\mu \right)$ and $Y_{EA/S}\left(\mu \right)$ courses exhibited distinct maxima at μ ≈ 0.5 h^–1^, corresponding to around 80% of $\mu_{max}$, while the $r_{EtOH}\left(\mu \right)$ and $Y_{EtOH/S}\left(\mu \right)$ courses steadily increased with the decreasing growth rate (Figures 5A and 5B). The decrease of the growth rate traced back to the temporally declining intracellular iron content which also effected the observed changes of the depicted rates and yields. Moderate iron limitation (at a higher growth rate) favored ester formation while severe iron limitation (at a lower growth rate) intensified ethanol synthesis.

This observation is in line with the hypothesis of ethyl acetate synthesis [10, 15, 30]: iron limitation renders the electron‐transport chain (ETC) less effective, the flux through the Krebs cycle is diminished, and accumulating acetyl‐CoA is diverted to synthesis of ethyl acetate via the Eat1 enzyme. Severe iron limitation in a late stage of cultivation reduces the ETC activity seriously which makes the metabolism mainly fermentative. Such a shift from respiratory to fermentative sugar metabolism has also been observed in *K. marxianus* DSM 5422 at rising ETC‐inhibitor levels [30]: yeast growth without significant metabolite formation at low inhibitor levels, some growth and ester synthesis at moderate inhibitor levels, and nearly no growth and ethanol formation at high inhibitor levels.

## 4 CONCLUDING REMARKS

Microbial synthesis of ethyl acetate as a bulk product is usually evaluated via the overall yield ($Y_{EA/S}$). However, there are additional parameters like the biomass‐specific rate of ester synthesis ($r_{EA}$) and the productivity of the process ($R_{EA}$) that should be considered for optimization and economic assessment of such processes. A recently developed balancing method enabled the quantitative comparison of ester synthesis by several yeast species at various induction modes in a high temporal resolution. The best results regarding synthesis of ethyl acetate were achieved under iron‐limited conditions with *K. marxianus* DSM 5422. Oxygen limitation also triggered synthesis of ethyl acetate but favored formation of ethanol over ethyl acetate.

Iron‐limited batch cultures are subject to a steadily changing cell metabolism; ongoing cell growth causes a temporally decreasing intracellular iron content which, in turn, diminishes the growth rate and modulates the ester synthesis. Moderate iron deficiency causes a high synthesis rate of ethyl acetate whereas severe iron deficiency decreases ester formation but favors ethanol production. The yield and rate of ester synthesis seem to exhibit a distinct maximum at a particular intracellular iron content. This knowledge can be used to develop an advanced fed‐batch or continuous process that sustains the intracellular iron content at an optimum level over an extended period.

The high potential of yeasts with GRAS status for synthesis of ethyl acetate enables development of industrial‐scale processes for converting sugar‐rich waste streams of the food industry into this ester. Such a waste is whey, arising in large amounts during cheese production. Whey allows for lactose production, but usage of remaining mother liquors is problematical. Value‐added production of ethyl acetate from such mother liquors could be an alternative to the currently practiced ethanol production. Some advantages argue for ester over ethanol production [30]: ethyl acetate exhibits a higher market price, the number of process stages is reduced, the process is quicker, product inhibition is avoided by ester stripping, and the stripping enables an energy‐efficient process‐integrated product recovery.

## CONFLICT OF INTEREST

The authors have declared no conflicts of interest.

## NOMENCLATURE

**Table:** 

Symbol	[Unit]	Description
$C_{AA,G}$	[g L^−1^]	Concentration of acetaldehyde in the gas at the gas‐line exit
$C_{EA,G}$	[g L^−1^]	Concentration of ethyl acetate in the gas at the gas‐line exit
$C_{EtOH,G}$	[g L^−1^]	Concentration of ethanol in the gas at the gas‐line exit
$C_{EtOH,L}$	[g L^−1^]	Concentration of ethanol in the cultivation medium
$C_{S}$	[g L^−1^]	Sugar concentration in the cultivation medium
$C_{VOC,G}$	[g L^−1^]	Concentration of a VOC in the gas at the gas‐line exit
$C_{X}$	[g L^−1^]	Biomass concentration in the cultivation medium given as dry weight
DO	[%]	Dissolved‐oxygen concentration related to the saturation concentration
$F_{G}$	[L h^−1^]	Gas flow leaving the bioreactor system (gas‐line exit)
$F_{G,R}$	[L h^−1^]	Gas flow leaving the bioreactor at headspace conditions
$K_{VOC,L/G}$	[L L^−1^]	Partition coefficient of a VOC between the liquid and gas in a bioreactor
$m_{AA}$	[g]	Mass of cumulatively synthesized acetaldehyde
$m_{EA}$	[g]	Mass of cumulatively synthesized ethyl acetate
$m_{EtOH}$	[g]	Mass of cumulatively synthesized ethanol
$m_{VOC}$	[g]	Mass of cumulatively synthesized VOC
$R_{AA}$	[g L^−1^h^−1^]	Volume‐specific rate of acetaldehyde synthesis
$R_{EA}$	[g L^−1^h^−1^]	Volume‐specific rate of ethyl acetate synthesis
$R_{EtOH}$	[g L^−1^h^−1^]	Volume‐specific rate of ethanol synthesis
$R_{VOC}$	[g L^−1^h^−1^]	Volume‐specific rate of VOC synthesis
RQ	[mol mol^−1^]	Respiratory quotient
$r_{AA}$	[g g^−1^h^−1^]	Biomass‐specific rate of acetaldehyde synthesis/degradation
$r_{EA}$	[g g^−1^h^−1^]	Biomass‐specific rate of ethyl acetate synthesis/degradation
$r_{EtOH}$	[g g^−1^h^−1^]	Biomass‐specific rate of ethanol synthesis/degradation
$r_{VOC}$	[g g^−1^h^−1^]	Biomass‐specific rate of VOC synthesis/degradation
*t*	[h]	Process time
$V_{L}$	[L]	Volume of the cultivation medium in the bioreactor
$x_{Fe}$	[μg g^−1^]	Intracellular iron content related to the biomass dry weight
$Y_{AA/S}$	[g g^−1^]	Overall yield of acetaldehyde
$Y_{EA/S}$	[g g^−1^]	Overall yield of ethyl acetate
$Y_{EA/S,max}$	[g g^−1^]	Maximum yield of ethyl acetate
$Y_{EtOH/S}$	[g g^−1^]	Overall yield of ethanol
$Y_{VOC/S}$	[g g^−1^]	Overall yield of a VOC
*Greek symbols*		
$\beta_{VOC}$	[−]	Relative retention of a VOC by the exhaust‐gas condenser
Δt	[h]	Time interval
μ	[h^−1^]	Specific growth rate
$\mu_{max}$	[h^−1^]	Maximum specific growth rate
τ	[h]	Control variable at integration

## Supporting information

Supplementary information
