# Supplementary material for: Synthesis of ethyl acetate from glucose by Kluyveromyces marxianus, Cyberlindnera jadinii and Wickerhamomyces anomalus depending on the induction mode
Source: Eng Life Sci. 2020 Dec 23;21(3-4):154–68. doi: 10.1002/elsc.202000048 (PMC7923572; doi:10.1002/elsc.202000048)
Supplement: Supplementary file 1 — Supplementary information [file ELSC-21-154-s001.pdf]

# Supporting information: Yields and rates of ester synthesis

## Synthesis of ethyl acetate from glucose by *Kluyveromyces marxianus*, *Cyberlindnera jadinii* and *Wickerhamomyces anomalus* depending on the induction mode

Andreas Hoffmann, Christian Kupsch, Thomas Walther, Christian Löser

Used symbols are listed in the main part of the work or are explained here in the text. References are labelled by [S...] and listed at the end of this Supporting information.

Reported yields of ethyl acetate in the literature were already summarized earlier (Table 2 in [S1]). Here, this review has been extended in many respects: (1) ester yields are given differentiated for various process conditions; (2) varied parameters for influencing the ester synthesis are indicated; (3) the trigger for inducing the ester synthesis is named (differentiated into intended and unintended induction); (4) the volume-specific as well as the biomass-specific synthesis rates are given; (5) the used cultivation system is characterized; and (6) the experimental procedure is evaluated regarding possible losses of ester during the process.

### Restrictions

In this Supporting information, only processes with ethyl acetate synthesis by growing cultures have been evaluated while processes using resting cells (e. g., described in [S2–S4]) have been ignored. All considered processes used sugar as a substrate, while processes based on ethanol (e. g., in [S2–S10]) or a mixture of ethanol and sugar (e. g., in [S11–S14]) were not evaluated. A further restriction concerns the microorganisms; only wild-type strains of yeasts were taken into account while bacteria and genetically engineered yeasts (e. g., in [S14–S16]) were excluded.

### Explanations

*Yeast strain* The list of ethyl acetate synthesizing microorganisms is restricted to yeasts and to wild-type strains although a growing number of publications deals with the formation of ethyl acetate by genetically modified yeasts and bacteria. The given yeast names are often not identical with the names used in the original reference, i. e., the naming is orientated on the current state of knowledge.

*Substrate* Synthesis of ethyl acetate has been studied based on diverse sugars and ethanol as well. The data tabulated below are restricted to processes based on sugars. The most frequently used sugar was glucose, but some studies were based on other hexoses such as fructose and galactose. Another often used raw material was whey consisting of lactose and some galactose (here quoted as lactose). Processes with ethanol as a substrate have been excluded from Table S1.

*Varied parameter* The third column of Table S1 gives information about the process parameter which was variegated in the considered study to take influence on the ester synthesis. However, such a parameter variation only occurred in part of the evaluated references.

*Trigger of ester synthesis* The synthesis of ethyl acetate in wild-type strains is induced by special cultivation conditions such as limitation of yeast growth by iron, copper and/or oxygen. Alternatively, ester synthesis can also be induced by partial inhibition of the electron-transport chain using specific inhibitors. Induction of ester synthesis occurred intentionally or accidentally. For this reason, it was differentiated between an intended and an unintended induction. For example, there are studies where ester synthesis had been induced by a lack of iron but, at the same time, oxygen became a limiting factor

as well (in this case, iron limitation was the intended trigger, while oxygen limitation was the unintended trigger). The risk of oxygen limitation develops at aerobic yeast cultivation in sugar-rich culture media without adequate mixing, e. g., at cultivation in shake flasks. Ethanol synthesis by Crabtree-negative yeasts may refer to oxygen limitation.

$Y_{EA/S}$ This parameter is the yield of ethyl acetate which is calculated from the grams of synthesized ester related to the grams of consumed sugar. The parameter was calculated for the total process or for the moment when the maximum amount of ester had been synthesized (in case the synthesized ester was consumed again by the yeasts). In case of absent sugar data, a complete sugar utilization was assumed. Values given in brackets were not stated directly in the cited literature but were calculated from the published data.

$Y_{EA/S}/Y_{EA/S,max}$ This parameter is the yield of ethyl acetate related to the possible maximum yield, given in percent. This maximum yield is obtained from stoichiometric considerations, when the sugar is assumedly exclusively converted into ethyl acetate without any yeast growth and by-product synthesis. Kruis [S16] named this $Y_{EA/S}$-$Y_{EA/S,max}$ ratio pathway efficiency. The $Y_{EA/S,max}$ value depends on the metabolic pathway and the used substrate. Here, synthesis of ethyl acetate from ethanol and acetyl-CoA via the Eat1 enzyme is assumed, where acetyl-CoA originates from a respiratory process, resulting in the following maximum yields: $Y_{EA/Glucose,max}$ = 0.489 g $g^{-1}$, $Y_{EA/Lactose,max}$ = 0.515 g $g^{-1}$, or $Y_{EA/EtOH,max}$ = 0.956 g $g^{-1}$. Values given in brackets were not stated directly in the cited literature but were calculated from the published data.

$R_{EA,max}$ This is a process parameter describing the maximum volume-specific rate of ester synthesis and is given in grams of formed ester per liter reaction volume and per hour. When this parameter is changing during the considered process, then the maximum value is given. Values in brackets were not stated directly in the cited literature but were calculated from the depicted data.

$r_{EA,max}$ This is a biological parameter describing the maximum biomass-specific synthesis rate of ethyl acetate and is given in grams of formed ester per gram of biomass dry weight and per hour. When this parameter is changing during the considered process, then the maximum value is given. Values in brackets were not stated directly in the cited literature but were calculated from depicted data.

*Cultivation system* The used cultivation system is shortly characterized. Cultivations have been performed in standing or shaken flasks or in stirred bioreactors. Culture flasks were fitted with cotton plugs, or the culture flasks were completely sealed with rubber plugs or screw cups (then referred as to ‘sealed’). Stirred bioreactors were operated in the batch mode (then simply named ‘bioreactor’) or in a continuous mode (then termed as ‘chemostat’). Cultivation systems were usually aerated, but in some cases purged with $N_2$ (for anaerobic conditions) or with pure $O_2$ (for a better oxygen transfer).

*Losses of ester?* Ethyl acetate is highly volatile and is therefore stripped from flushed cultivation systems (e. g., from aerated bioreactors) or leaves the system by diffusion (e. g., from cotton-plugged shake flasks). Such losses of ethyl acetate during the process have to be taken into account for precise balancing of ester synthesis. The entry ‘yes’ means, that such a loss occurred without adequate consideration, while ‘no’ stands for processes in completely sealed systems without such losses or for losses which were quantified. The term ‘probably’ labels processes where a loss of ester seems plausible. The superscripts give more specified information on this subject.

*Reference* The references are given in form of [S...] and are listed at the end of this Supporting information.

**Table S1.** Yields and rates of ester synthesis from the literature depending on the cultivated strain and the cultivation conditions (detailed explanation above).

| Yeast species, Strain | Substrate | Varied parameter | Trigger of ester synthesis | | $Y_{EA/S}$ [$g_{EA}$ $g_S^{-1}$] | $Y_{EA/S}/Y_{EA/S,max}$ [%] | $R_{EA,max}$ [$g_{EA}$ $L^{-1}h^{-1}$] | $r_{EA,max}$ [$g_{EA}$ $g_X^{-1}h^{-1}$] | Cultivation system | Losses of ester | Reference |
|---|---|---|---|---|---|---|---|---|---|---|---|
| | | | intended | unintended | | | | | | | |
| *Cyberlindnera fabianii* CBS 5640 | Glucose | Fe availability | Fe limitation | - | 0.16 | 32.7 | (0.82) | (0.26) | Aerated chemostat | no [a)] | [S17] |
| *Cyberlindnera fabianii* CBS 5640 | Glucose | Fe availability | - | - | 0.001 | 0.2 | - | - | Aerated chemostat | no [a)] | [S17] |
| *Cyberlindnera jadinii* ATCC 9950 | Glucose | $O_2$ availability | Fe limitation | $O_2$ limitation | (0.09) | (18) | (0.79) | - | Shaken sealed flask | no [b)] | [S3] |
| *Cyberlindnera jadinii* ATCC 9950 | Glucose | $O_2$ availability | Fe + $O_2$ limitation | - | (0.03 - 0.18) | (6 - 37) | - | - | Shaken flask | probably [c)] | [S3] |
| *Cyberlindnera jadinii* ATCC 9950 | Glucose | Fe availability | heavy Fe limitation | $O_2$ limitation | (0.25 - 0.26) | (51 - 53) | - | - | Shaken sealed flask | no [b)] | [S18] |
| *Cyberlindnera jadinii* ATCC 9950 | Glucose | Fe availability | medium Fe limitation | $O_2$ limitation | (0.09) | (18) | - | - | Shaken sealed flask | no [b)] | [S18] |
| *Cyberlindnera jadinii* ATCC 9950 | Glucose | Fe availability | slight Fe limitation | $O_2$ limitation | (0.04) | (8) | - | - | Shaken sealed flask | no [b)] | [S18] |
| *Cyberlindnera jadinii* ATCC 9950 | Glucose | Fe availability | Fe limitation | $O_2$ limitation | (0.24) | (49) | (0.12) | - | Shaken sealed flask | no [b)] | [S18] |
| *Cyberlindnera jadinii* ATCC 9950 | Glucose | Fe availability | - | $O_2$ limitation | (0.00) | (0) | - | - | Shaken sealed flask | no [b)] | [S18] |
| *Cyberlindnera jadinii* ATCC 9950 | Glucose | - | Fe limitation | ? $O_2$ limitation | (0.27) | (56) | (0.23) | (0.15) | Aerated system | yes [d)] | [S19] |
| *Cyberlindnera jadinii* CECT 1956 | Glucose | Fe availability | Fe limitation | - | (0.105) | (21.5) | (0.59) | (0.07) | Aerated chemostat | no [a)] | [S17] |
| *Cyberlindnera jadinii* CECT 1956 | Glucose | Fe availability | - | - | 0.000 | 0.0 | - | - | Aerated chemostat | no [a)] | [S17] |
| *Cyberlindnera jadinii* DSM 2361 | Glucose | Limiting factor | - | - | 0.000 | 0.0 | 0.00 | 0.000 | Aerated bioreactor | no [a)] | this work |
| *Cyberlindnera jadinii* DSM 2361 | Glucose | Limiting factor | Fe limitation | - | 0.028 | 5.7 | 0.11 | 0.034 | Aerated bioreactor | no [a)] | this work |
| *Cyberlindnera jadinii* DSM 2361 | Glucose | Limiting factor | $O_2$ limitation | - | 0.021 | 4.3 | 0.45 | 0.053 | Aerated bioreactor | no [a)] | this work |
| *Kluyveromyces lactis* CBS 739 | Lactose | Culture system | Fe limitation | $O_2$ limitation | (0.19) | (36) | - | - | Shaken sealed flask | no [b)] | [S20] |
| *Kluyveromyces lactis* CBS 739 | Lactose | Culture system | Fe limitation | $O_2$ limitation | 0.18 | 35 | 0.68 | - | $O_2$-purged flask | no a) | [S20] |
| *Kluyveromyces lactis* CBS 743 | Lactose | Culture system | Fe limitation | $O_2$ limitation | (0.14) | (28) | - | - | Shaken sealed flask | no [b)] | [S20] |
| *Kluyveromyces lactis* CBS 743 | Lactose | Culture system | Fe limitation | $O_2$ limitation | 0.18 | 35 | 0.59 | - | $O_2$-purged flask | no [a)] | [S20] |
| *Kluyveromyces lactis* CBS 2359 | Glucose | Fe availability | Fe limitation | - | 0.04 | 8.2 | (0.23) | (0.046) | Aerated chemostat | no [a)] | [S17] |
| *Kluyveromyces lactis* CBS 2359 | Glucose | Fe availability | - | - | 0.000 | 0.0 | - | - | Aerated chemostat | no [a)] | [S17] |
| *Kluyveromyces marxianus* | Lactose | $O_2$ availability | Fe limitation | - | (0.013) | (2.6) | - | - | Aerated bioreactor | yes [d)] | [S12] |
| *Kluyveromyces marxianus* | Lactose | $O_2$ availability | Fe limitation | - | (0.019) | (3.6) | (0.02) | - | Aerated bioreactor | yes [d)] | [S12] |
| *Kluyveromyces marxianus* | Lactose | $O_2$ availability | Fe + $O_2$ limitation | - | (0.004) | (0.8) | (0.02) | - | Aerated chemostat | yes [d)] | [S12] |
| *Kluyveromyces marxianus* | Lactose | $O_2$ availability | Fe limitation | - | (0.039) | (7.6) | (0.20) | - | Aerated chemostat | yes [d)] | [S12] |
| *Kluyveromyces marxianus* | Lactose | C/N ratio | Fe limitation | - | (0.01 - 0.08) | (2.4 - 14.5) | - | - | Aerated chemostat | yes [d)] | [S12] |
| *Kluyveromyces marxianus* | Lactose | - | Fe + $O_2$ limitation | - | (0.007) | (1.4) | (0.02) | (0.008) | Aerated chemostat | yes [d)] | [S13] |
| *Kluyveromyces marxianus* (21 strains) | Lactose | Strain | Fe limitation | $O_2$ limitation | (0.01 - 0.17) | (2 - 33) | - | - | Shaken sealed flask | no [b)] | [S20] |
| *Kluyveromyces marxianus* (10 strains) | Lactose | Strain | Fe limitation | $O_2$ limitation | 0.13 - 0.23 | 25 - 45 | 0.37 - 0.91 | - | $O_2$-purged flask | no [a)] | [S20] |
| *Kluyveromyces marxianus* CBS 6556 | Glucose | Sugar + strain | - | ? $O_2$ limitation | (0.245) | (50.1) | - | - | Shaken flask | probably [c)] | [S15] |
| *Kluyveromyces marxianus* CBS 6556 | Fructose | Sugar + strain | - | ? $O_2$ limitation | (0.185) | (37.8) | - | - | Shaken flask | probably [c)] | [S15] |
| *Kluyveromyces marxianus* CBS 6556 | Galactose | Sugar + strain | - | ? $O_2$ limitation | (0.190) | (38.9) | - | - | Shaken flask | probably [c)] | [S15] |
| *Kluyveromyces marxianus* CBS 6556 | Glucose | - | Fe limitation | - | (0.186) | (38.0) | 0.80 | - | Aerated bioreactor | no [a)] | [S21] |

**Table S1.** Continuation 1.

| Yeast species, Strain | Substrate | Varied parameter | Trigger of ester synthesis | | $Y_{EA/S}$ [$g_{EA}$ $g_S^{-1}$] | $Y_{EA/S}/Y_{EA/S,max}$ [%] | $R_{EA,max}$ [$g_{EA}$ $L^{-1}h^{-1}$] | $r_{EA,max}$ [$g_{EA}$ $g_X^{-1}h^{-1}$] | Cultivation system | Losses of ester | Reference |
|---|---|---|---|---|---|---|---|---|---|---|---|
| | | | intended | unintended | | | | | | | |
| *Kluyveromyces marxianus* DSM 5422 | Lactose | Medium + Fe avail. | - | - | < 0.01 | < 2 | - | - | Aerated bioreactor | no a) | [S22] |
| *Kluyveromyces marxianus* DSM 5422 | Lactose | Medium + Fe avail. | Fe limitation | - | 0.25 | 49 | 5.33 | (0.52) | Aerated bioreactor | no a) | [S22] |
| *Kluyveromyces marxianus* DSM 5422 | Lactose | Medium + Fe avail. | - | - | < 0.01 | < 2 | - | - | Aerated bioreactor | no a) | [S22] |
| *Kluyveromyces marxianus* DSM 5422 | Lactose | Medium + Fe avail. | Fe limitation | - | 0.23 | 45 | 2.94 | - | Aerated bioreactor | no a) | [S22] |
| *Kluyveromyces marxianus* DSM 5422 | Lactose | Medium + Fe avail. | - | - | < 0.01 | < 2 | - | - | Aerated bioreactor | no a) | [S22] |
| *Kluyveromyces marxianus* DSM 5422 | Lactose | Medium + Fe avail. | Fe limitation | - | 0.07 | 14 | 0.86 | - | Aerated bioreactor | no a) | [S22] |
| *Kluyveromyces marxianus* DSM 5422 | Lactose | Medium + Fe avail. | - | - | < 0.01 | < 2 | - | - | Aerated bioreactor | no a) | [S22] |
| *Kluyveromyces marxianus* DSM 5422 | Lactose | Medium + Fe avail. | Fe limitation | - | 0.19 | 37 | 1.69 | - | Aerated bioreactor | no a) | [S22] |
| *Kluyveromyces marxianus* DSM 5422 | Lactose | Limiting factor | Fe limitation | - | 0.25 | 49 | 2.70 | 0.83 | Aerated bioreactor | no a) | [S23] |
| *Kluyveromyces marxianus* DSM 5422 | Lactose | Limiting factor | Cu limitation | - | 0.05 | 9 | 0.98 | 0.09 | Aerated bioreactor | no a) | [S23] |
| *Kluyveromyces marxianus* DSM 5422 | Lactose | Limiting factor | Zn limitation | - | 0.01 | 2 | 0.27 | 0.04 | Aerated bioreactor | no a) | [S23] |
| *Kluyveromyces marxianus* DSM 5422 | Lactose | Fe availability | ± Fe limitation | - | 0.00 - 0.23 | 0 - 45 | - | - | Sealed shaken flask | no b) | [S24] |
| *Kluyveromyces marxianus* DSM 5422 | Lactose | Dilution rate | - | - | 0.01 - 0.05 | 2 - 10 | (0.01 - 0.56) | 0.000 - 0.031 | Aerated chemostat | no a) | [S24] |
| *Kluyveromyces marxianus* DSM 5422 | Lactose | Dilution rate | Fe limitation | - | 0.05 - 0.15 | 10 - 29 | (0.25 - 0.49) | 0.084 - 0.215 | Aerated chemostat | no a) | [S24] |
| *Kluyveromyces marxianus* DSM 5422 | Lactose | Fe availability | ± Fe limitation | - | 0.05 - 0.22 | 9 - 43 | (0.20 - 1.51) | 0.020 - 0.437 | Aerated chemostat | no a) | [S24] |
| *Kluyveromyces marxianus* DSM 5422 | Lactose | Temperature | Fe limitation | - | 0.198 | 38.4 | 1.61 | 0.54 | Aerated bioreactor | no a) | [S25] |
| *Kluyveromyces marxianus* DSM 5422 | Lactose | Temperature | (Fe limitation) | - | 0.002 | 0.4 | 0.05 | 0.01 | Aerated bioreactor | no a) | [S25] |
| *Kluyveromyces marxianus* DSM 5422 | Lactose | Temperature | Fe limitation | - | 0.289 | 56.2 | 4.23 | 0.67 | Aerated bioreactor | no a) | [S25] |
| *Kluyveromyces marxianus* DSM 5422 | Lactose | Gas flow | Fe limitation | - | 0.213 - 0.219 | 41.4 - 42.5 | 2.49 - 2.95 | 0.26 - 0.40 | Aerated bioreactor | no a) | [S26] |
| *Kluyveromyces marxianus* DSM 5422 | Lactose | Fe availability | heavy Fe limitation | - | 0.265 | 51.4 | 2.45 | 0.490 | Aerated bioreactor | no a) | [S27] |
| *Kluyveromyces marxianus* DSM 5422 | Lactose | Fe availability | medium Fe limitation | - | 0.239 | 46.3 | 3.13 | 0.337 | Aerated bioreactor | no a) | [S27] |
| *Kluyveromyces marxianus* DSM 5422 | Lactose | Fe availability | slight Fe limitation | - | 0.113 | 21.9 | 1.95 | 0.174 | Aerated bioreactor | no a) | [S27] |
| *Kluyveromyces marxianus* DSM 5422 | Lactose | $O_2$ availability | $O_2$ limitation | - | 0.004 | 0.8 | 0.05 | (0.008) | Aerated bioreactor | no a) | [S28] |
| *Kluyveromyces marxianus* DSM 5422 | Lactose | $O_2$ availability | $O_2$ excluded | - | 0.001 | 0.2 | 0.005 | (0.002) | $N_2$-purged bioreactor | no a) | [S28] |
| *Kluyveromyces marxianus* DSM 5422 | Lactose | ETC inhibitor | ± Carboxin | - | 0.000 - 0.136 | 0.0 - 26.4 | - | - | Sealed shaken flask | no b) | [S28] |
| *Kluyveromyces marxianus* DSM 5422 | Lactose | ETC inhibitor | ± Antimycin A | - | 0.000 - 0.037 | 0.0 - 7.2 | - | - | Sealed shaken flask | no b) | [S28] |
| *Kluyveromyces marxianus* DSM 5422 | Lactose | ETC inhibitor | ± Cyanide | - | 0.001 - 0.098 | 0.2 -19.0 | (0.00 - 0.07) | (0.00 - 0.17) | Sealed shaken flask | no b) | [S28] |
| *Kluyveromyces marxianus* DSM 5422 | Glucose | Sugar + strain | - | ? $O_2$ limitation | (0.188) | (38.4) | - | - | Shaken flask | probably c) | [S15] |
| *Kluyveromyces marxianus* DSM 5422 | Fructose | Sugar + strain | - | ? $O_2$ limitation | (0.170) | (34.8) | - | - | Shaken flask | probably c) | [S15] |
| *Kluyveromyces marxianus* DSM 5422 | Galactose | Sugar + strain | - | ? $O_2$ limitation | (0.218) | (44.7) | - | - | Shaken flask | probably c) | [S15] |

**Table S1.** Continuation 2.

| Yeast species, Strain | Substrate | Varied parameter | Trigger of ester synthesis | | $Y_{EA/S}$ [$g_{EA}$ $g_S^{-1}$] | $Y_{EA/S}/Y_{EA/S,max}$ [%] | $R_{EA,max}$ [$g_{EA}$ $L^{-1}h^{-1}$] | $r_{EA,max}$ [$g_{EA}$ $g_X^{-1}h^{-1}$] | Cultivation system | Losses of ester | Reference |
|---|---|---|---|---|---|---|---|---|---|---|---|
| | | | intended | unintended | | | | | | | |
| *Kluyveromyces marxianus* DSM 5422 | Glucose | Fe availability | Fe limitation | - | (0.136) | (27.8) | (1.08) | (0.157) | Aerated chemostat | no [a)] | [S17] |
| *Kluyveromyces marxianus* DSM 5422 | Glucose | Fe availability | - | - | 0.000 | 0.0 | - | - | Aerated chemostat | no [a)] | [S17] |
| *Kluyveromyces marxianus* DSM 5422 | Glucose | Limiting factor | - | - | 0.024 | 4.9 | 0.18 | 0.038 | Aerated bioreactor | no [a)] | this work |
| *Kluyveromyces marxianus* DSM 5422 | Glucose | Limiting factor | Fe limitation | - | 0.182 | 37.2 | 0.86 | 0.502 | Aerated bioreactor | no [a)] | this work |
| *Kluyveromyces marxianus* DSM 5422 | Glucose | Limiting factor | $O_2$ limitation | - | 0.042 | 8.6 | 0.56 | 0.145 | Aerated bioreactor | no [a)] | this work |
| *Kluyveromyces marxianus* NCYC 143 | Glucose | Fe + $O_2$ availability | heavy Fe limitation | ? $O_2$ limitation | (0.015) | (3.0) | - | - | Shaken flask | probably [c)] | [S8] |
| *Kluyveromyces marxianus* NCYC 143 | Glucose | Fe + $O_2$ availability | - | $O_2$ limitation | (0.0015) | (0.3) | - | - | Shaken flask | probably [c)] | [S8] |
| *Kluyveromyces marxianus* NCYC 143 | Glucose | Fe + $O_2$ availability | $O_2$ excluded | - | 0.00 | 0.0 | - | - | Sealed shaken flask | no [b)] | [S8] |
| *Lachancea kluyveri* Y708 | Glucose | - | - | - | 0.080 | 16.3 | (0.64) | (0.117) | Aerated bioreactor | no [a)] | [S29] |
| *Wickerhamomyces anomalus* | Glucose | - | - | heavy $O_2$ limitation | (0.07) | (14) | (0.10) | - | Standing flask | probably [c)] | [S30] |
| *Wickerhamomyces anomalus* | Glucose | $O_2$ availability | - | heavy $O_2$ limitation | 0.123 | 25.1 | - | - | Sealed standing flask | no [b)] | [S31] |
| *Wickerhamomyces anomalus* | Glucose | $O_2$ availability | $O_2$ excluded | - | 0.005 | 1.1 | - | - | Sealed standing flask | no [b)] | [S31] |
| *Wickerhamomyces anomalus* | Glucose | growth factors | - | heavy $O_2$ limitation | (0.13- 0.18) | (27 - 38) | (0.00 - 0.06) | - | Standing flask | probably [c)] | [S5] |
| *Wickerhamomyces anomalus* | Glucose | growth factors | - | slight $O_2$ limitation | (0.074) | (15) | (0.09) | (0.041) | Shaken flask | probably [c)] | [S5] |
| *Wickerhamomyces anomalus* CECT 10590 | Glucose | $O_2$ availability | heavy $O_2$ limitation | - | (0.017) | (3.4) | - | - | Standing flask | probably [c)] | [S32] |
| *Wickerhamomyces anomalus* CECT 10590 | Glucose | $O_2$ availability | - | $O_2$ limitation | (0.104) | (21.2) | - | - | Shaken flask | probably [c)] | [S32] |
| *Wickerhamomyces anomalus* DSM 6766 | Glucose | $O_2$ availability | - | - | 0 | 0 | 0 | 0 | Aerated chemostat | no [a)] | [S14] |
| *Wickerhamomyces anomalus* DSM 6766 | Glucose | $O_2$ availability | $O_2$ limitation | - | 0.064 | 13.1 | 0.064 | - | Aerated chemostat | no [a)] | [S14] |
| *Wickerhamomyces anomalus* DSM 6766 | Glucose | Fe availability | Fe limitation | - | 0.17 | 34.8 | (1.16) | (0.276) | Aerated chemostat | no [a)] | [S17] |
| *Wickerhamomyces anomalus* DSM 6766 | Glucose | Fe availability | - | - | 0.000 | 0.0 | - | - | Aerated chemostat | no [a)] | [S17] |
| *Wickerhamomyces anomalus* DSM 6766 | Glucose | Limiting factor | - | - | 0.000 | 0.0 | 0.00 | 0.000 | Aerated bioreactor | no [a)] | this work |
| *Wickerhamomyces anomalus* DSM 6766 | Glucose | Limiting factor | Fe limitation | - | 0.046 | 9.4 | 0.16 | 0.044 | Aerated bioreactor | no [a)] | this work |
| *Wickerhamomyces anomalus* DSM 6766 | Glucose | Limiting factor | $O_2$ limitation | - | 0.053 | 10.8 | 0.63 | 0.112 | Aerated bioreactor | no [a)] | this work |
| *Wickerhamomyces ciferrii* CBS 111 | Glucose | - | Fe limitation | $O_2$ limitation | (0.130) | (26.5) | (0.16) | - | Shaken flask | probably [c)] | [S14] |
| *Wickerhamomyces anomalus* CBS 1984 | Glucose | $O_2$ availability | - | - | - | - | - | 0.00005 | Aerated bioreactor | yes [d)] | [S33] |
| *Wickerhamomyces anomalus* CBS 1984 | Glucose | $O_2$ availability | $O_2$ limitation | - | - | - | - | 0.001 | Aerated bioreactor | yes [d)] | [S33] |

[a)] = stripping handled adequately, e.g., by analyzing the stripped ester in the exhaust gas
[b)] = stripping not occurring due to cultivation in a completely sealed system
[c)] = assumed losses because of uncertain sealing (e.g., by cotton stoppers) or non-reported sealing
[d)] = stripping handled in a wrong way (e.g., by using an exhaust-gas condenser for retention of stripped ester)

## References


[S1] Löser, C., Urit, T., Bley, T., Perspectives for the biotechnological production of ethyl acetate by yeasts. *Appl. Microbiol. Biotechnol.* 2014, *98,* 5397–5415.

[S2] Tabachnick, J., Joslyn, M. A., Formation of esters by yeast. II. Investigations with cellular suspensions of *Hansenula anomala*. *Plant. Physiol.* 1953, *28*, 681–692.

[S3] Armstrong, D. W., Martin, S. M., Yamazaki, H., Production of ethyl acetate from dilute ethanol solutions by *Candida utilis*. *Biotechnol. Bioeng.* 1984, 26, 1038–1041.

[S4] Armstrong, D. W., Martin, S. M., Yamazaki, H., Production of acetaldehyde from ethanol by *Candida utilis*. *Biotechnol. Lett.* 1984, *6*, 183–188.

[S5] Tabachnick, J., Joslyn, M. A., Formation of esters by yeast. I. The production of ethyl acetate by standing surface cultures of *Hansenula anomala*. *J. Bacteriol.* 1953, *65*, 1–9.

[S6] Armstrong, D. W., Selective production of ethyl acetate by *Candida utilis*. *ACS Symp.* 1986, *317,* 254–265.

[S7] Bol, J., Knol, W., ten Brik, B., Optimization of the production of ethyl acetate from ethanol by *Hansenula anomala*. *Dechema Monographs* 1987, *105*, 235–236.

[S8] Willetts, A., Ester formation from ethanol by *Candida pseudotropicalis*. *Antonie van Leeuwenhoek* 1989, *56*, 175–180.

[S9] Corzo, G., Revah, S., Christen, P., Effect of oxygen on the ethyl acetate production from continuous ethanol stream by *Candida utilis* in submerged cultures. *Develop. Food Sci.* 1995, *37B*, 1141–1154.

[S10] Christen, P., Domenech, F., Páca, J., Revah, S., Evaluation of four *Candida utilis* strains for biomass, acetic acid and ethyl acetate production from ethanol. *Bioresour. Technol.* 1999, *68,* 193–195.

[S11] Yong, F. M., Lee, K. H., Wong, H. A., The production of ethyl acetate by soy yeast *Saccharomyces rouxii* NRRL Y-1096. *J. Food Technol.* 1981, *16*, 177–184.

[S12] Kallel-Mhiri, H., Engasser, J. M., Miclo, A., Continuous ethyl acetate production by *Kluyveromyces fragilis* on whey permeate. *Appl. Microbiol. Biotechnol*. 1993, *40*, 201–205.

[S13] Kallel-Mhiri, H., Miclo, A., Mechanism of ethyl acetate synthesis by *Kluyveromyces fragilis*. *FEMS Microbiol. Lett.* 1993, *111*, 207–212.

[S14] Kruis, A. J., Levisson, M., Mars, A. E., van der Ploeg, M., et al., Ethyl acetate production by the elusive alcohol acetyltransferase from yeast. *Metab. Eng.* 2017, *41*, 92–101.

[S15] Löbs, A.-K., Lin, J.-L., Cook, M., Wheeldon, I., High throughput, colorimetric screening of microbial ester biosynthesis reveals high ethyl acetate production from *Kluyveromyces marxianus* on C5, C6, and C12 carbon sources. *Biotechnol. J.* 2016, 11, 1–8.

[S16] Kruis, A. J., Towards biobased ethyl acetate and beyond. Identification and application of the elusive Eat1 enzyme. Dissertation, Wageningen University, Wageningen, the Netherlands 2018.

[S17] Kruis, A. J., Mars, A. E., Kengen, S. W. M., Borst, J. W., et al., Alcohol acetyltransferase Eat1 is located in yeast mitochondria. *Appl. Environ. Microbiol.* 2018, *84* (*19*), e01640-18, 1–11.

[S18] Armstrong, D. W., Yamazaki, H., Effect of iron and EDTA on ethyl acetate accumulation in *Candida utilis*. *Biotechnol. Lett.* 1984, *6*, 819–824.

[S19] Williams, R. E., Armstrong, D. W., Murray, W. D., Welsh, F. W., Enzyme and whole cell production of flavor and fragrance compounds. *Ann. New York Acad. Sci.* 1988, *542*, 406–412.

[S20] Löser, C., Urit, T., Nehl, F., Bley, T., Screening of *Kluyveromyces* strains for the production of ethyl acetate: design and evaluation of a cultivation system. *Eng. Life Sci.* 2011, *11*, 369–381.

[S21] Löbs, A.-K., Engel, R., Schwartz, C., Flores, A., et al., CRISPR-Cas9-enabled genetic disruptions for understanding ethanol and ethyl acetate biosynthesis in *Kluyveromyces marxianus*. *Biotechnol. Biofuels* 2017, *10* (*164*), 1–14.

[S22] Urit, T., Löser, C., Wunderlich, M., Bley, T., Formation of ethyl acetate by *Kluyveromyces marxianus* on whey: studies of the ester stripping. *Bioprocess Biosyst. Eng.* 2011, *34*, 547–559.

[S23] Urit, T., Stukert, A., Bley, T., Löser, C., Formation of ethyl acetate by *Kluyveromyces marxianus* on whey during aerobic batch cultivation at specific trace-element limitation. *Appl. Microbiol. Biotechnol*. 2012, *96*, 1313–1323.

[S24] Löser, C., Urit, T., Förster, S., Stukert, A., Bley, T., Formation of ethyl acetate by *Kluyveromyces marxianus* on whey during aerobic batch and chemostat cultivation at iron limitation. *Appl. Microbiol. Biotechnol.* 2012, *96*, 685–696.

[S25] Urit, T., Li, M., Bley, T., Löser, C., Growth of *Kluyveromyces marxianus* and formation of ethyl acetate depending on temperature. *Appl. Microbiol. Biotechnol.* 2013, *97*, 10359–10371.

[S26] Urit, T., Manthey, R., Bley, T., Löser, C., Formation of ethyl acetate by *Kluyveromyces marxianus* on whey: influence of aeration and inhibition of yeast growth by ethyl acetate. *Eng. Life Sci.* 2013, *13*, 247–260.

[S27] Löser, C., Urit, T., Stukert, A., Bley, T., Formation of ethyl acetate from whey by *Kluyveromyces marxianus* on a pilot scale. *J. Biotechnol.* 2013, *163*, 17–23.

[S28] Löser, C., Urit, T., Keil, P., Bley, T., Studies on the mechanism of synthesis of ethyl acetate in *Kluyveromyces marxianus* DSM 5422. *Appl. Microbiol. Biotechnol.* 2015, *99*, 1131–1144.

[S29] Møller, K., Christensen, B., Förster, J., Piškur, J., Nielsen, J., Olsson, L., Aerobic glucose metabolism of *Saccharomyces kluyveri*: growth, metabolite production, and quantification of metabolic fluxes. *Biotechnol. Bioeng.* 2002, *77*, 186–193.

[S30] Gray, W. D., Initial studies on the metabolism of *Hansenula anomala* (Hansen) Sydow. *Am. J. Bot.* 1949, *36*, 475–480.

[S31] Davies, R., Falkiner, E. A., Wilkinson, J. F., Peel, J. L. Ester formation by yeasts 1. Ethyl acetate formation by *Hansenula* species. *Biochem. J.* 1951, *49*, 58–61.

[S32] Rojas, V., Gil, J. V., Piñaga, F., Manzanares, P. Studies on acetate ester production by non-*Saccharomyces* wine yeasts. *Int. J. Food Microbiol.* 2001, *70*, 283–289.

[S33] Fredlund E., Blank, L. M., Schnürer, J., Sauer, U., et al., Oxygen and glucose-dependent regulation of central carbon metabolism in *Pichia anomala*. *Appl. Environ. Microbiol.* 2004, *70*, 5905–5911.
